# Research on Intelligent Identification of Pivoting Center and Smooth Processing of Test Data for Flying Flexible Joint

**DOI:** 10.3389/fnbot.2021.666285

**Published:** 2021-04-29

**Authors:** Yue-bing Wen, Jian-ping Tan

**Affiliations:** ^1^School of Mechanical and Electrical Engineering, Central South University, Changsha, China; ^2^Hunan Industry Polytechnic, Changsha, China

**Keywords:** pivot center shift, machine vision, intelligent identification, image processing, smooth processing, calculation of pivoting center

## Abstract

In this paper, a method of intelligent identification and data smooth processing of flying flexible joint pivoting center based on machine vision is proposed. The intelligent identification is realized by the following process: first of all the geometric center of the two markers attached to the flying body is located on a straight line at a certain angle to the center-line of the measured pivoting body, secondly then continuous image sampling is carried out by industrial camera when the marker swings with the pivoting body, and image data is transmitted through a data interface to an industrial computer, Finally the image processing module de-noises the image, removes the background and locates the markers to obtain the plane coordinates of the markers in the coordinate system of the test system. The data smooth of obtained coordinates is carried outby Matlab software including the following steps: the coordinates of the mark points detected based on machine vision are optimized to obtain the smooth curve by fitting of the parabola and arc. Then the coordinates of the points on the curve are used to optimize the coordinates of the marked points from measurement. The optimized coordinate values are substituted into the calculation module of pivoting center, so the average pivoting center of the sampling interval of two images is calculated according to the mathematical model to approach the instantaneous pivoting center during the motion of the pivoting body. The result processing module displays and records the curve of pivoting center shift directly and effectively. Finally, it is validated by simulation and experiments that the precision of pivoting center measured by such measuring system is ~0.5%.

## Introduction

During flight, the deviation of the flying pivoting center will affect the actual clearance of the nozzle swing, the change of the relationship between the stroke of the cylinder and the swing angle, and the change of the power arm, etc. (Kearney and Moss, 1993; Ellis et al., 1997; Boury et al., 2008; Carney et al., 2020). And the actual deviating rules between axial and radial direction of pivoting center for precision swing is also an important basis for overall design and decision-making (Alam et al., 2019; Cui and Wu, 2019), therefore, it is very important to accurately measure the pivoting center data of flight body of precision swing to ensure the precision of thrust vector control of aircraft. During swinging (Liu et al., 2019), the flexible joint of flying body should move along the smooth curve, but because of the error of positioning, the research on the swing center recognition technology of flexible joint of flying body belongs to the state secret, so there are few published literatures. The measurement methods of flexible joint position of flight body are divided into two types: contact measurement and non-contact measurement. The contact measurement method is based on displacement sensor. Yang first proposed the concept of the swing center and the angle of the flexible joint. According to the characteristics of the intersection of the instantaneous axis plane and the geometric center line plane of the flexible joint, the pendulum center is obtained (Yang, 1985). Ma et al. have given a measurement scheme based on high precision calibration of zero position bar. The measurement of the swing angle of flexible joint can be completed by computer control (Ma and Yang, 2011). Most of the above contact measurement methods use displacement sensors to measure the flight body pendulum center indirectly, and its measurement accuracy needs to be improved.

In view of the many defects of contact method, more and more non-contact measurement methods are proposed. Zhu puts the self-emitting target marking plate on the side of flexible joint, and uses linear CCD devices to measure the swing angle (Zhu, 1993). Zhang et al. uses the infrared camera around the flexible joint to capture the position of the feature points, and uses the conic surface fitting method to obtain the axis and swing angle of the flexible joint. The shape error of the conical surface of the flexible joint will affect the measurement accuracy (Zhang et al., 2010). Seely, combines GaAs infrared laser diode with displacement sensitive detector to measure the swing angle by using two sets of experimental data at different angles.

Hugh pointed out that the selection of flexible joint materials will have different effects on the pendulum center and the swing angle (Reynolds and Morrow, 1975). Robert proposed that the center, angle and axial displacement of flexible joints play an important role in the process of thrust vector control.

Aiming at solving the problems above, this paper presents a research on shift measurement of flexible joint based on intelligent recognition of machine vision and data smoothing.

**Figure 1:**
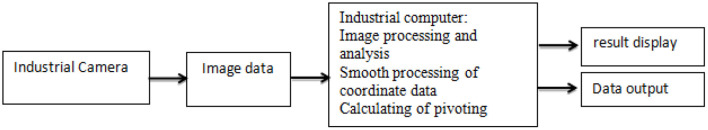
System structure.

The system test flow is as shown in Figure 1, the industrial camera begins to collect the original image data after the system begins to work, and the original data is transmitted to the data processing program on the industrial computer through the USB standard data interface. The data processing program processes and analyzes the image to obtain the coordinates of markers at each time. Then by using the Matlab, the optimized coordinates can be obtained by smooth processing the measured coordinates, and then the pivoting center shift state of the flexible joint will be obtained by the calculation program. Finally, the measurement results are output through the man-machine interface and data files.

## Materials and Methods

The original measurement method of the system is based on the displacement sensor. Three displacement sensors are installed on the flexible joint. The center of pendulum is calculated by the increment of the swing angle and the corresponding relationship between the displacement sensor and the displacement sensor. The instantaneous pendulum center is determined by the swing angle, so the data of the flexible joint center swing may not be “real.”

### System Structure

**Figure 2:**
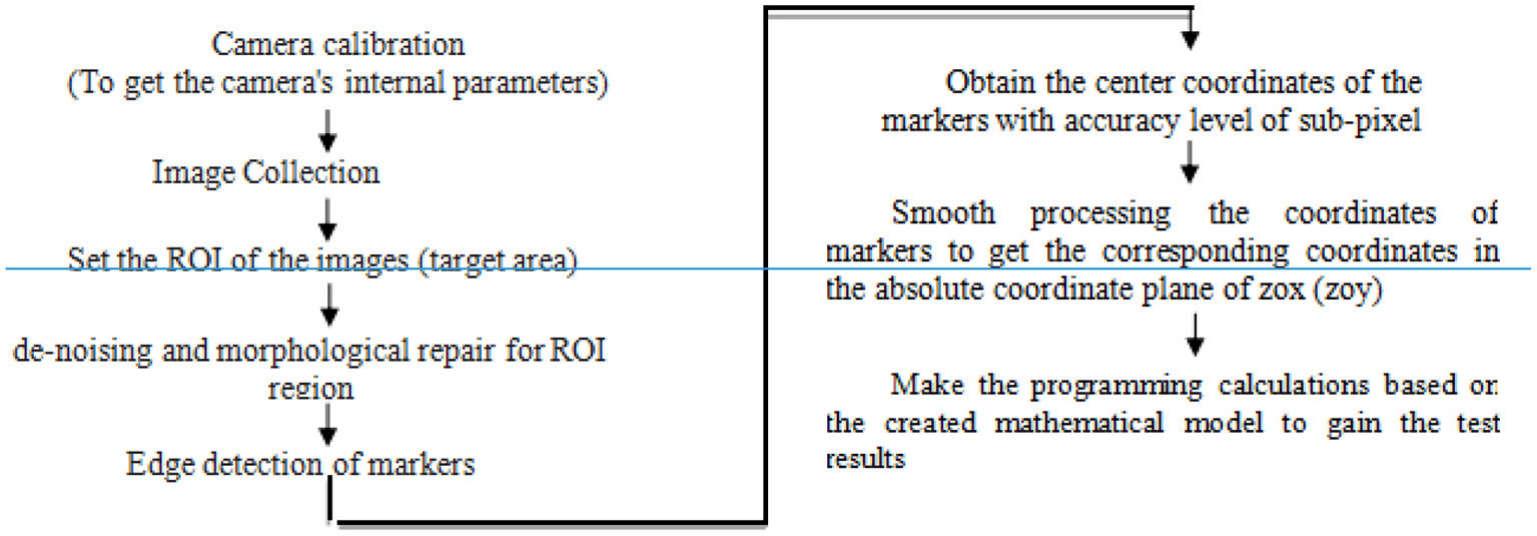
Overall technical flow chart.

The overall technical flow is shown in Figure 2. Before the system begins to work, the internal parameters such as pixel ratio and distortion coefficient of the machine vision system are calibrated. Then, the camera begins to collect the images, and transmit them to the industrial computer through the data interface for image processing. The image processing flow is as below: (1) According to the moving range of each marker on the images, make ROI set for all images. (2) Morphological repair and denoising filtering for ROI regions of images. (3) Detect the edge and extract the center point of the markers on the images to get the coordinates of the markers with accuracy level of sub-pixel, and then make the smooth processing to these coordinates, finally, calculate the pivoting center shift state of flexible joint on industrial computer based on the relation and mathematical model of each coordinate system.

### Selection of Industrial Camera and Its Lens

By analyzing the test data of pivoting center shift of flexible joint, it can be seen that the higher frequency of original data acquisition will lead to the excessive detection noise, and the proper reduction of the frequency will not distort the curve of the detection results (Tao et al., 2020; Zhang, 2020; Moru and Borro, 2021). Analysis shows that the high precision detection of pivoting center shift of flexible joint can be achieved when the image acquisition frequency reaches 21 Hz (Pohanka et al., 2018; Damirchi et al., 2019; Marina et al., 2020; Zhou et al., 2020).

Considering the technical requirements of the pivoting center shift detection system of flexible joints, the industrial camera of DMK 31AU03 produced by German Imaging Source Company is preliminarily selected as the image acquisition element of the system. The specific parameters of the camera are shown in Table 1.

**Table 1:** Main technical parameters of DMK 31AU03.

**Items**	**Spec**	**Items**	**Spec**
Effective pixel	1024 × 768	Data interface	USB2.0
Image sensor	1/3 " Sony CCD, Sequential scanning	Pixel dimensions	Horizontal: 4.65 μm, Vertical: 4.65 μm
Frame rate	30 frames/s	Size	50.6mm × 50.6mm × 50mm

The preliminary analysis shows that the distance from the lens of the detection system to the target object is about 330 mm. Considering the requirement of the object distance and the quality reliability, the lenses of fixed focal length (M0814-MP) from the Japanese CBC company can meet the needs of the detection system. The specifications are shown in Table 2 (Zhao et al., 2019; Yang, 2020).

**Table 2:** Specifications of M0814-MP.

**Items**	**Spec**	**Items**	**Spec**
Model	M0814-MP	Back focal length (mm)	13.10
Target size	2/3″	Interface	C-Interface
Focal length (mm)	8	Minimum Object Distance (M.O.D) (m)	0.1 m
Maximum imaging dimensions (mm)	8.8 × 6.6 (Φ11)	(F-stop) range of aperture	F1.4 F16C
Size (mm)	Φ33.5 × 28.2	Resolution (Center/Edge)	100 lpm

### Realization of System Function

Based on DMK 31AU03, the structure of detection system is that the industrial camera can be connected directly to an industrial computer via a USB data line. The models of data acquisition, smooth processing and pivoting center calculation on the industrial computer can complete the processing of the original image data to output the pivoting center shift state of the flexible joint at each time with the interface of the human-computer and the data file (Zhang, 2000). And the system can realize output of on-line detection results.

### Calculation of System Performance Index

By analyzing the moving range of the measured body and considering the effective pixel length-width ratio of the camera, we can see that view field of 200 (mm) × 160 (mm) can meet all requirements for detecting the moving range, so horizontal and vertical resolution Hl, Hv for single pixel can be as:

$$\begin{array}{l} Hl=\frac{200}{1024}\approx 0.195\left(mm\right)Hv=\frac{150}{768}\approx 0.195\left(mm\right) \end{array}$$

To achieve sub-pixel image positioning accuracy, the horizontal, and vertical image positioning accuracy of the markers (μ) is 0.0195 mm.

According to the relevant image experiments, it takes about 20 ms to complete a medium-complex image processing on the industrial computer, which means 50 frames of image processing per second. The data processing speed can keep up with the data acquisition speed for this system with acquisition speed of 30 frames of original data per second, so the system can display the pivoting center shift state in real time without the result output delay.

As mentioned above, the technical performance index of this hardware scheme is shown in Table 3.

**Table 3:** Estimation of main performance parameters based on DMK 31AU03 machine vision system.

**Items**	**Spec**	**Items**	**Spec**
Swing angular velocity	0 7.5°/s	Test system accuracy	Axial 1.5 mm, Transversal 0.3 mm (10%)
Maximum swing angular acceleration	25r ad/s^2^	Repeat positioning accuracy	5%
Maximum swing angle	±8°	Operating temperature	0 35°
Frequency	0.2 0.5 HZ	Output delay	Real-time output, no delay
Swing mode	Swing in plane		

Table 3 shows that the system can fully meet the project requirements.

### Smooth Processing of Test Data

#### Smooth Processing Introduction

The commonly used curve smooth processing methods (least square method, energy method, spring-back method) are all to calculate the minimum value of the objective functions, which has consistent basic ideas (Fua and Sander, 1992). The objective function is the weighted average of the deviation of the value points and the smooth processing results, and all value points are used for smooth processing.

In addition to the use of regular curves during smooth processing, the situation is often encountered: a smooth curve is needed to be constructed with some discrete calculated or test data, which is passing through or close to these discrete points, and such constructed curve is called a fitting curve (interpolation curve) (Levin, 1998, 2004). In practical applications, the general principle of choosing curve smooth processing method is depending on the characteristics of practical applications. In particular, it can be considered in two ways: If the given data is small quantity and strictly accurate, the interpolation method should be chosen; If the given data is from a large number of results of tests or statistics, which are not necessary to be strictly passed through, but play a qualitative control role, then the method of data fitting should be chosen (Dey et al., 2004).

Because the data used in this paper are a large number of test points, a smooth curve is constructed by least square method for these data, and the actual pivoting center of flexible joint is calculated from the curve.

#### Smooth Processing of Test Data of Markers

##### Raw Data Processing

The dynamic data (each set of data contains the X coordinates and Z coordinates of 100 pcs of point A and point B) obtained from on-site camera are processed as follows: Considering the interference error, the average value of every 5 points is calculated for the original data to obtain 20 sets of processing data.

##### Smooth Processing of Test Data of Markers

The coordinates of the point A and point B after raw data processing are represented as (*u*_*A*_, *v*_*A*_) and (*u*_*B*_, *v*_*B*_), then the parabola equation and arc equation are used to smooth processing of these 20 sets of coordinates, so the corresponding coordinates of point A and point B after fitting are expressed as (*x*_*A*_, *z*_*A*_) and (*x*_*B*_, *z*_*B*_), the equations obtained from Matlab are as following:

(1) $$\begin{array}{l} \text{Parabola fitting equation of point A}:z=0.00042032x^{2}\text{-}1.6305x+1492.6; \end{array}$$

(2) $$\begin{array}{l} \text{Parabola fitting equation of point B}:z=0.0006349x^{2}\text{-}1.833x+1293; \end{array}$$

(3) $$\begin{array}{l} \text{Arc fitting equation of point A:}x^{2}+z^{2}\text{-}5029.3x\text{-}4418.4z+550.1=0; \end{array}$$

(4) $$\begin{array}{l} \text{Arc fitting equation of point B:}x^{2}+z^{2}\text{-}5067.9x\text{-}4473.5z+465.3=0. \end{array}$$

### The Calculation of Pivoting Center Coordinates After Smooth Processing

The coordinates of marker A (*x*_*A*_, *z*_*A*_) and marker B (*x*_*B*_, *z*_*B*_) after smooth fitting are substituted into the pivoting center calculation program to obtain new coordinates.

**Figure 3:**
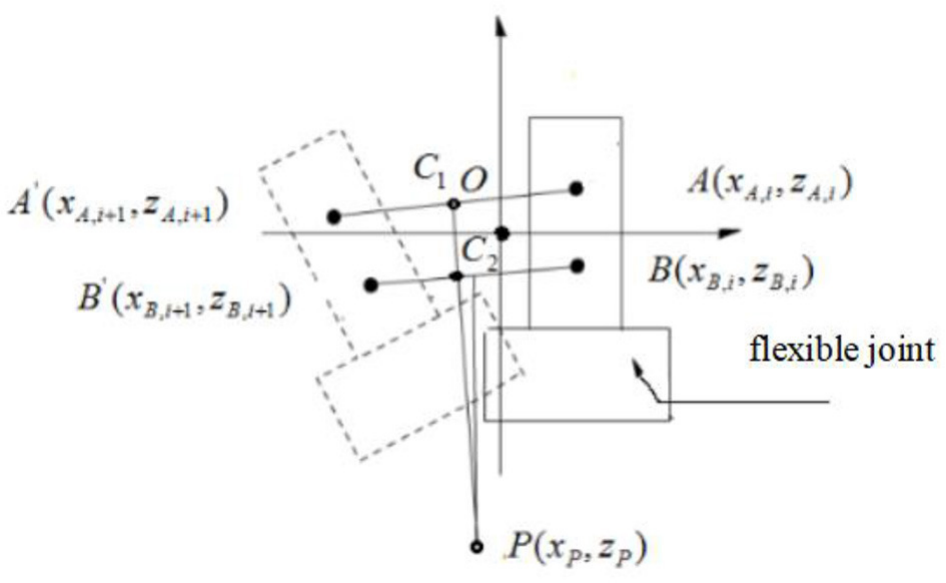
Schema of calculation of pivoting center.

The calculation steps of pivoting center coordinates are as follows:

In Figure 3, point O is the position of the marker A in the static state, that is, the origin of the XOZ plane.

Let the coordinates of the marker A and B in the XOZ plane be (*x*_*A*__,*i*_, *z*_*A*__,*i*_) and (*x*_*B*__*i*_, *z*_*B*__,*i*_) after round i of smooth processing.

Then after round i+1 of smooth processing, marker A and B is recorded as A' and B', and then their coordinates in XOZ plane can be presented as (*x*_*A*__,*i*+1_, *z*_*A*__,*i*+1_), (*x*_*B*__,*i*+1_, *z*_*B*__,*i*+1_).

C_1_ and C_2_ are the midpoint of AA' and BB', respectively.

The C_1_P and C_2_P are the middle vertical lines of AA' and BB', and the intersection point P of the two vertical lines is the average pivoting center of round i and round i+1 of image acquisition.

Let the coordinates of point C_1_ and C_2_ be (*x*_*C*__,1_, *z*_*C*__,1_) and (*x*_*c*_,_2_, *z*_*c*_,_2_), then:

(5) $$\begin{array}{l} {x_{C}}_{{\;}_{1}}=\frac{x_{A,i}+x_{A,i+1}}{2}{z_{C}}_{{\;}_{1}}=\frac{{z_{A,}}_{i}+z_{A,i+1}}{2} \end{array}$$

(6) $$\begin{array}{l} {x_{C}}_{{\;}_{2}}=\frac{x_{B,i}+x_{B,i+1}}{2}{z_{C}}_{{\;}_{2}}=\frac{z_{B,i}+z_{B,i+1}}{2} \end{array}$$

The slopes of straight line AA' and BB' (K_1_,K_2_) can be presented as, respectively:

(7) $$\begin{array}{l} K_{1}=\frac{z_{A,i}-z_{A,i+1}}{{x_{A,}}_{i}-x_{A,i+1}}K_{2}=\frac{z_{B,i}-z_{B,i+1}}{x_{B,i}-x_{B,i+1}} \end{array}$$

Since the straight line C_1_P and C_2_P are perpendicular to straight line AA' and BB', respectively, the slopes of straight line C_1_P and C_2_P (K_3_, K_4_) are:

(8) $$\begin{array}{l} K_{3}=-\frac{1}{K_{1}}=\frac{{x_{A,}}_{i}-x_{A,i+1}}{z_{A,i+1}-z_{A,i}}K_{4}=-\frac{1}{K_{2}}=\frac{x_{B,i}-x_{B,i+1}}{z_{B,i+1}-z_{B,i}} \end{array}$$

Then the equations of straight line C_1_P and C_2_P are:

(9) $$\begin{array}{l} \text{Straightline}{\text{C}}_{1}\text{P}:\text{z}\text{-}{\text{z}}_{\text{c}1}={\text{K}}_{3}\left(\text{x}-{\text{x}}_{\text{c}1}\right) \end{array}$$

(10) $$\begin{array}{l} \text{Straightline}{\text{C}}_{2}\text{P}:\text{z}\text{-}{\text{z}}_{\text{c}2}={\text{K}}_{4}\left(\text{x}-{\text{x}}_{\text{c}2}\right) \end{array}$$

If *z*_*A*_ equals to *z*′_*A*_ or *z*_*B*_ equals to *z*′_*B*_, that is, straight line *AA*′ or *BB*′ is horizontal, then the equation of *C*_1_*P* or *C*_2_*P* can be presented as:

(11) $$\begin{array}{l} x=\frac{x_{A,i}+x_{A,i+1}}{2}orx=\frac{x_{B,i}+x_{B,i+1}}{2} \end{array}$$

According to 2–9, 2–10, 2–11, the coordinate of test point P(*x*_*p*_, *z*_*p*_) can be obtained as:

(12) $$\begin{array}{l} x_{P}=\frac{z_{C_{2}}-z_{C_{1}}+K_{4}\times x_{C_{2}}-K_{3}\times x_{C_{1}}}{K_{4}-K_{3}} \end{array}$$

(13) $$\begin{array}{l} z_{P}=\frac{K_{3}\left(z_{C_{2}}-z_{C_{1}}+K_{4}\times z_{C_{2}}-K_{3}\times z_{C_{1}}\right)}{K_{4}-K_{3}}\\ \;-K_{3}\times x_{C_{1}}+z_{C_{1}} \end{array}$$

According to above calculation process, the coordinate of first pivoting center (*x*_*p*_^1^, *z*_*p*_^1^) can be calculated with substitution of coordinates of marker A and B after round 1 and 2 smoothing fitting [(*x*_*A*__,1_, *z*_*A*__,1_) and (*x*_*B*__,*1*_, *z*_*B*__,1_), (*x*_*A*__,2_, *z*_*A*__,2_), and (*x*_*B*__,2_, *z*_*B*__,2_)] into (Equations 2–12). In the same way, the coordinates of all pivoting centers can be calculated.

## Results

### The Deviation of Coordinates of Mark Point

**Figure 4:**
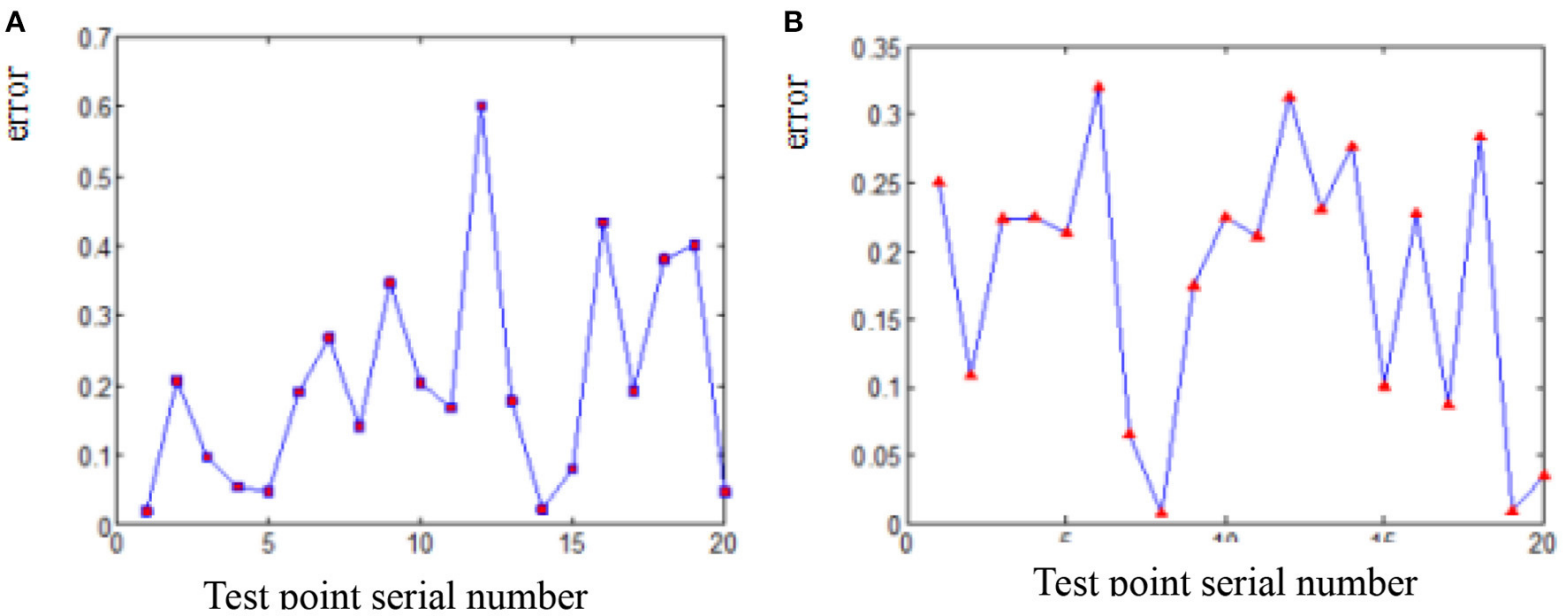
Coordinate error before and after smooth processing of marker A. **(A)** Parabola smoothing fitting. **(B)** Arc smoothing fitting.

**Figure 5:**
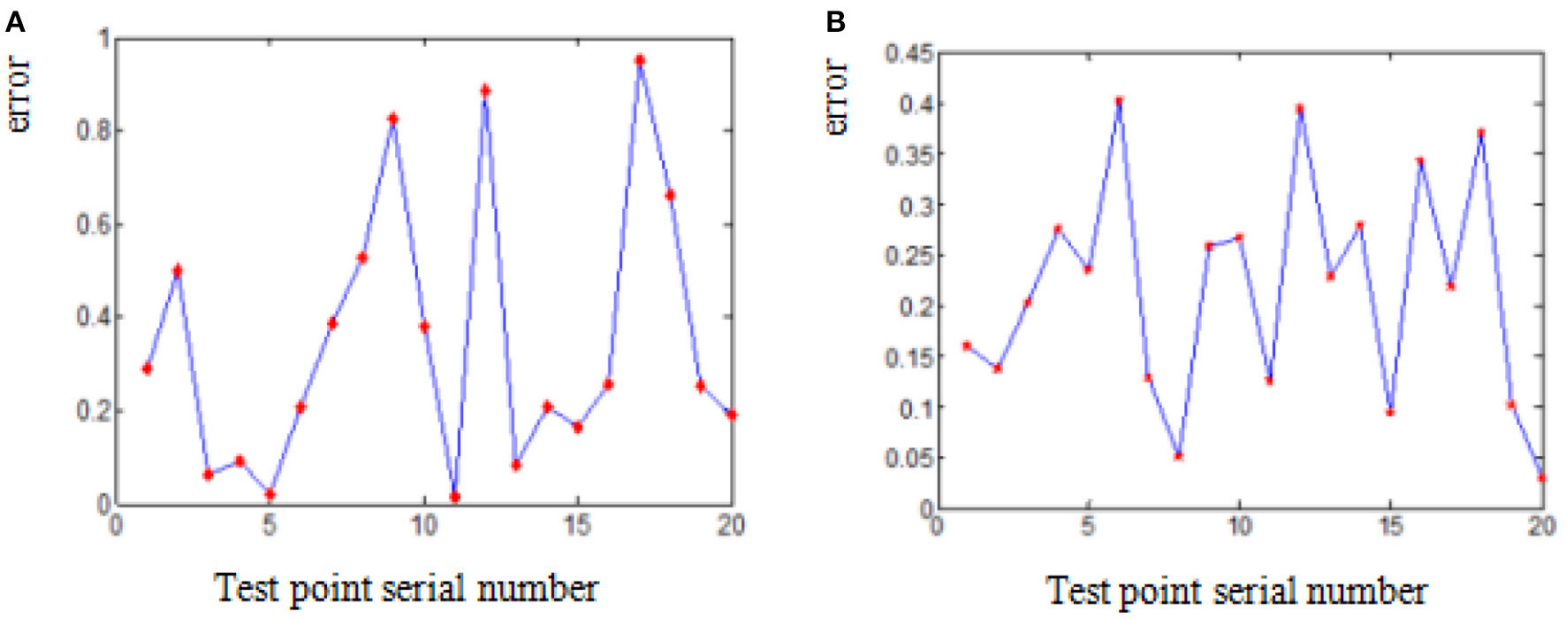
Coordinate error before and after smooth processing of Marker B. **(A)** Parabola smoothing fitting. **(B)** Arc smoothing fitting.

The deviation of coordinates of point A and B before and after smooth processing of parabola fitting and arc fitting (hereinafter abbreviated as “coordinate error”) is shown in Figures 4, 5 (the error of each point on the figures is expressed in absolute value). It can be seen from the diagrams that the error of arc smoothing fitting is slightly smaller than that of parabola smoothing fitting for marker A and B of swing body.

### Coordinates Comparison of Pivoting Centers Before and After Smooth Processing

**Figure 6:**
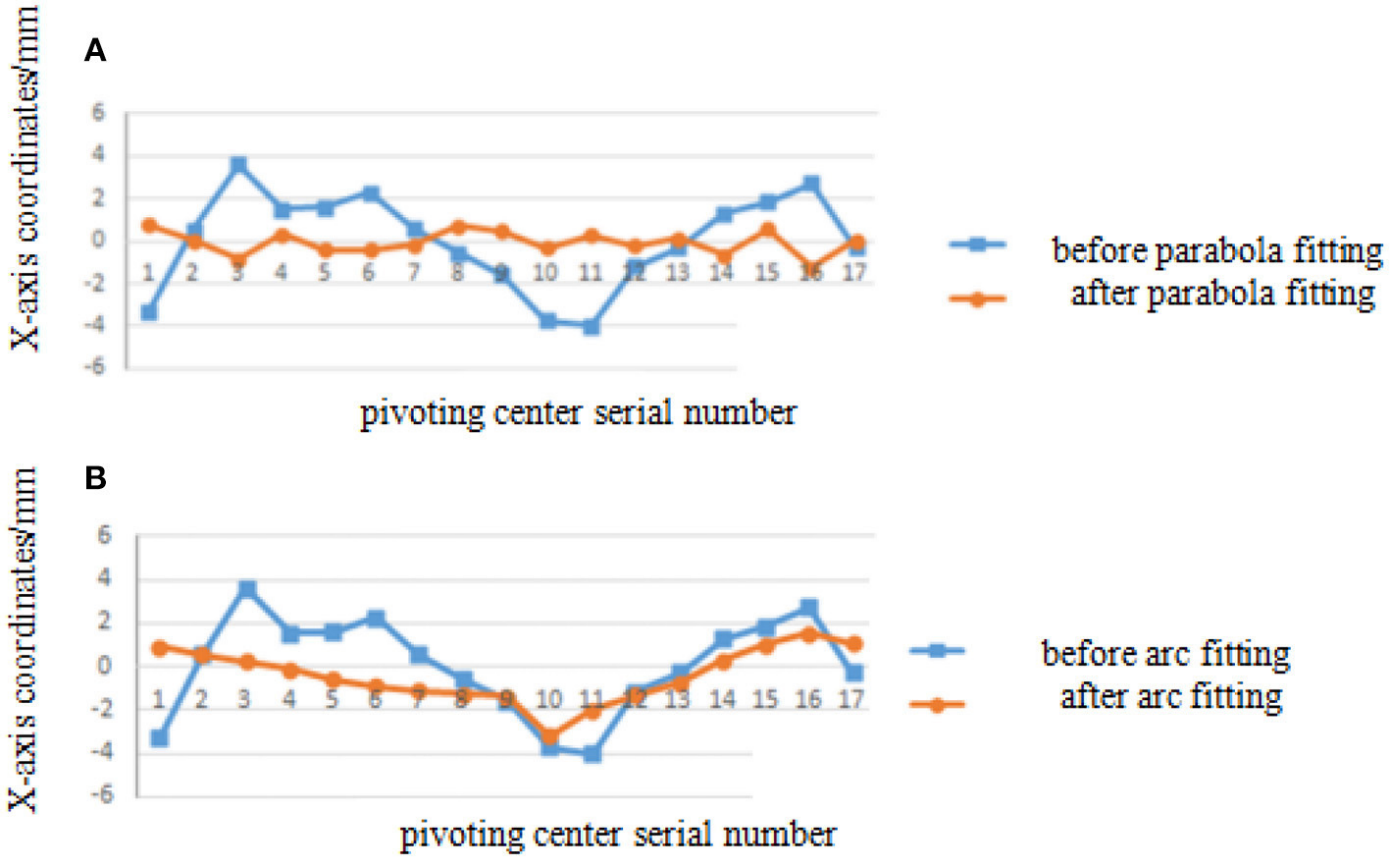
Comparison of X-axis coordinates before/after parabola fitting and arc fitting. **(A)** Diagram before/after parabola fitting. **(B)** Diagram before/after arc fitting.

**Figure 7:**
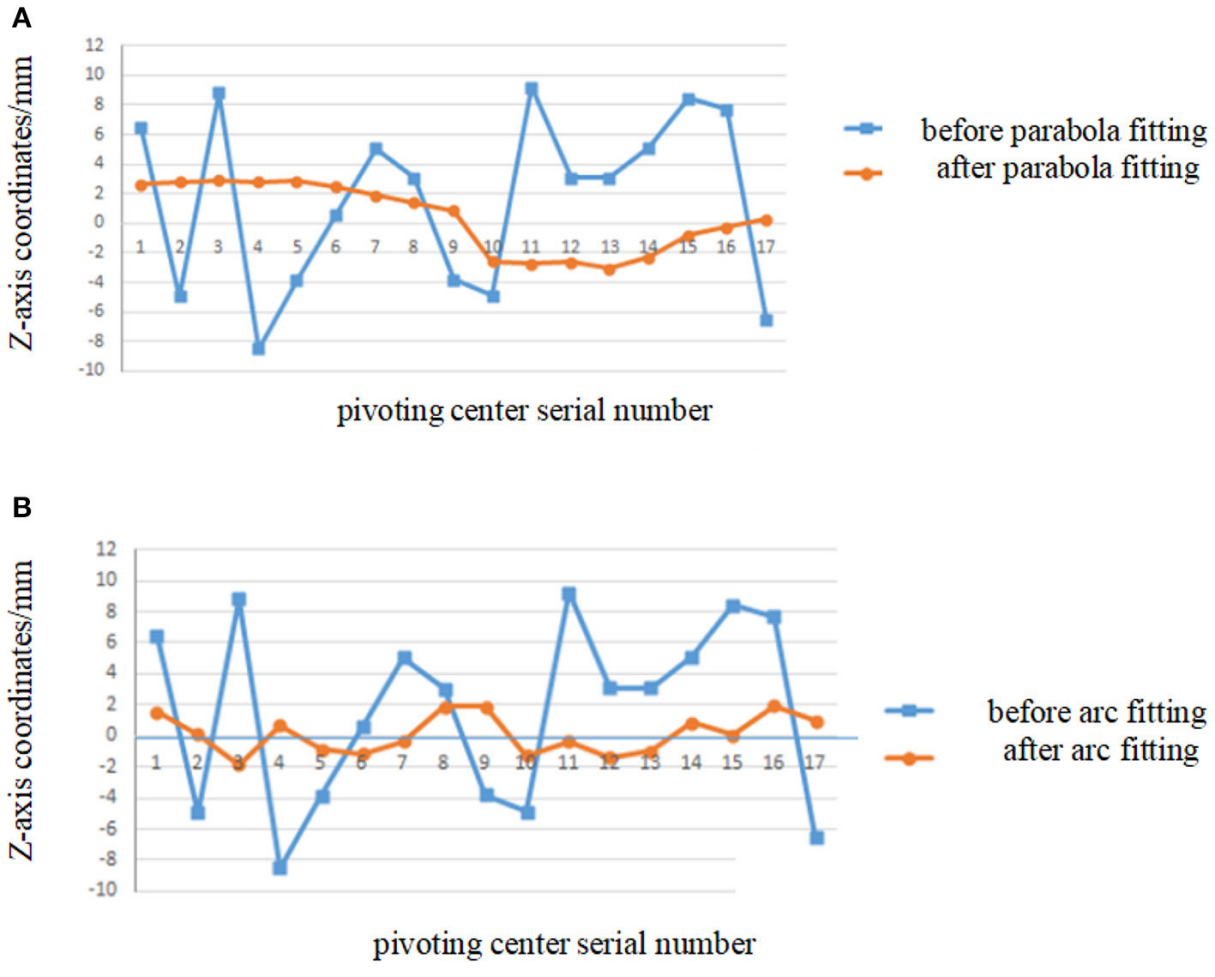
Comparison of Z-axis coordinates before/after parabola fitting and arc fitting. **(A)** Diagram before/after parabola fitting. **(B)** Diagram before/after arc fitting.

Step 1: Make raw data processing to the coordinates of marker A and B obtained from image acquisition.
Step 2: Make smooth processing with parabola fitting and arc fitting for these data.
Step 3: Substitute the fitted coordinates (Step 2) into pivoting center calculation program to obtain first two groups of pivoting center coordinates (Delete the first and last groups of data from above-mentioned 20 groups of data, in total, 17 pcs of pivoting center coordinates can be obtained from the remained 18 groups of data because one pivoting center coordinate can be obtained from two consecutive groups).
Step 4: Substitute the coordinates from Step 1 directly into pivoting center calculation program to obtain second two groups of pivoting center coordinates.
Step 5: Put them in the diagram: Figure 6 shows the comparison curve of X-direction coordinates after parabola fitting and arc fitting, and Figure 7 shows the comparison curve of Z-direction coordinates after parabola fitting and arc fitting.

It can be seen from Figures 6, 7 that the pivoting center shift along the X axis and the Z axis is more compliant after parabola fitting of the marker coordinates and the shift amplitude decreases obviously: the maximum amplitude reduces from 4 to 1.2 mm at X-axis and reduces from 8.2 to 2.2 mm at Z-axis.

It can be seen from Figures 6, 7 that for arc fitting, the shift amplitude of pivoting center coordinates reduces obviously, too, but the compliance is not as good as that of parabola fitting.

Table 4 shows that the maximum difference of coordinates of marker A & B and the maximum swing amplitudes of pivoting center at both X-axis and Z-axis before/after parabola fitting and arc fitting. Table 4 also shows that the swing amplitudes of pivoting center of flexible body are obviously reduced after both fitting. And although the maximum error after arc fitting is smaller than that after parabola fitting, the swing amplitude and compliance after parabola fitting are better than that after arc fitting.

**Table 4:** The maximum error and swing amplitude of pivoting center of marker A/B for parabola fitting and arc fitting.

**Smooth processing method**	**Phase**	**Maximum swing amplitude at X-axis/mm**	**Maximum swing amplitude at Z-axis/mm**	**Maximum error/mm**		**Compliance**
				***A***	***B***	
Parabola fitting	Before	4	8.2	0.60	0.95	Better
	After	1.2	2.2			
Arc fitting	Before	4	8.2	0.32	0.40	Sawteeth
	After	3.2	3.2			

### Experiments

#### Definition and Transformation of Test System Coordinate System

**Figure 8:**
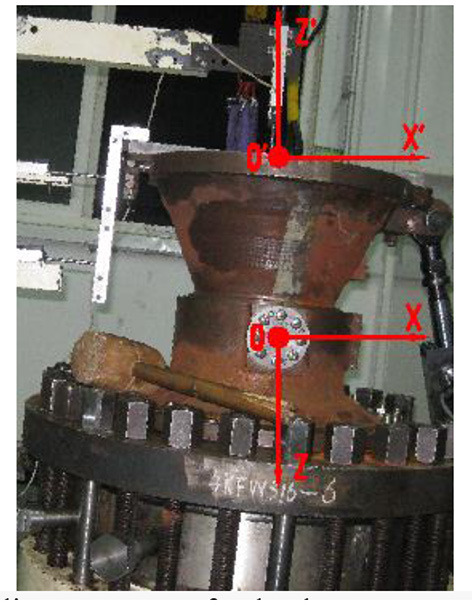
Definition of plane coordinate system for both test systems (New, XOZ; Original, X'O'Z').

Figure 8 is a rocket nozzle actuating device in a mechanical plant, the pivoting center coordinates are measured by the method described above in this paper and the original displacement sensor test system, respectively. The plane coordinate system (XOZ in Figure 6) is used for new test system described in this paper. The origin is the theoretical pivoting center. The horizontal right is positive direction of X-axis and the vertical downward is the positive direction of Z-axis.

The plane coordinate system (X'O'Z') is used for original displacement sensor test system (Figure 8). The origin is in the center of the upper datum plane, horizontal right is the positive direction of the X-axis, vertical upward is the positive direction of the Z-axis.

So when comparing two groups of test results, the test results from original displacement sensor test system are transformed from plane coordinate system X'O'Z' to XOZ (Yang, 1985).

#### Test Results

**Figure 9:**
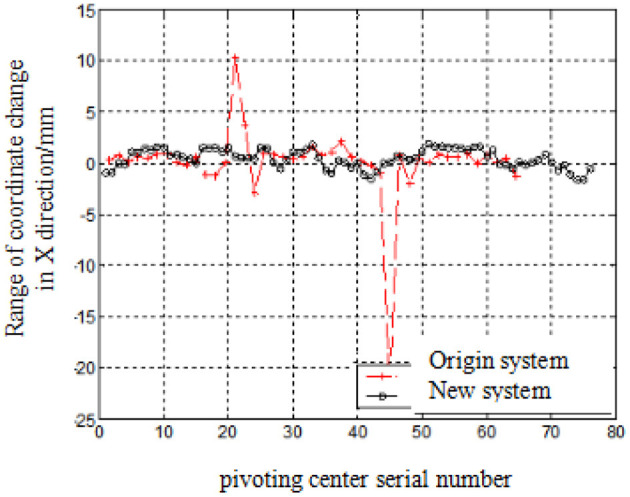
Test results at X-axis.

**Figure 10:**
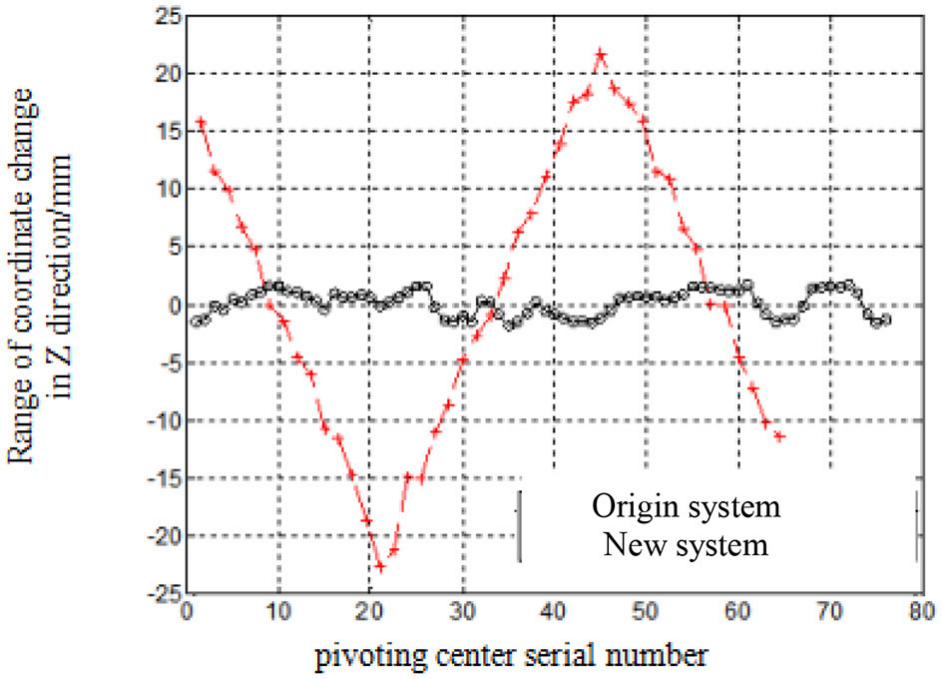
Test results at Z-axis.

Figures 9, 10 shows the range of coordinate change of pivoting center coordinates from machine vision intelligent identification test system (New system) and original displacement sensor test system (Original system) (the pressure curve is sine wave).

It can be seen that most calculated results from new system are in the range of ±8 mm at X-axis, but bigger range for those from original system. The error at Z-axis for new system is ~15 mm deviated from actual position, in the range of ±5 mm, but ±25 mm for original system.

## Discussion

This paper presents a research on shift measurement of flexible joint based on intelligent recognition of machine vision and data smoothing. This research combines the latest research results of machine vision and precision testing technology, and has the following advantages comparing with displacement sensor detection technology: the detection method is non-contact visual measurement, which reduces the requirement of mechanical reference accuracy in the test system; The automatic calibration of the industrial camera system is realized by the diameter size of the circular marker, which simplifies the initialization process after the system is reinstalled. In addition to the hardware optimization such as improving installation accuracy and camera accuracy, one of the key techniques in this study is to reduce the test error of pivoting center by data processing method, and propose a method of smoothing the measuring point coordinates and calculating the pivoting center to eliminate the interference of installation and test errors.

Table 5 shows the median error and its range of both test results of pivoting center coordinates for new system and original system.

**Table 5:** Precision contrast for machine vision intelligent identification test system and original displacement sensor test system.

**Test system**	**Error type**	**Spec**
Machine vision intelligent identification test system	Error range at X-axis	±1.5 mm
	Error range at Z-axis	±1.8 mm
Original displacement sensor test system	Error range at X-axis	±5 mm
	Error range at Z-axis	±20 mm

It can be seen from Table 5 that:

It can be seen from Table 5 that the X direction of the center test results of the mark machine vision system fluctuates within −1.5 to 1.5 mm, and the Z direction fluctuates within −1.8 to +1.8 mm;

The results of the pendulum center test of the displacement sensor test system fluctuate in one direction about 20 mm, and the other direction is mainly random error, within −5 to 5 mm.

Based on the research methods of machine vision intelligent recognition and test data smoothing, the latest research progress of artificial intelligence recognition and data smoothing is integrated.

## Conclusions

Compared with the existing technology, it has the following beneficial technical and economic effects:

(1) The intelligent identification of the mark points can be completed by collecting data from a single industrial camera, which reduces the link of error caused by contact in the traditional test process.
(2) The detection method is non-contact visual measurement, which reduces the requirement of the testing system on the precision of mechanical reference.
(3) The industrial camera system is calibrated automatically by the diameter of circular marker, which simplifies the initialization process after the system is reinstalled.
(4) By comparing the arc fairing fitting and parabola fairing fitting, it is concluded that although the maximum error of arc fitting process is smaller than that of parabola fitting, the swing amplitude of the pendulum detected after parabola fitting is smaller and the flexibility is better.
(5) Through the test, it can be seen that the research method of machine vision intelligent recognition and test data smoothing is compared with the original displacement sensor detection method. The method in this paper has a great advantage in the measurement error and accuracy of the measurement coordinates.

## Data Availability Statement

The original contributions presented in the study are included in the article/Supplementary Material, further inquiries can be directed to the corresponding author/s.

## Author Contributions

Yb-w was responsible for writing papers, simulation, and experiments. Jp-T was responsible for instructing ideas and directions, and checking papers. Both authors contributed to the article and approved the submitted version.

## Conflict of Interest

The authors declare that the research was conducted in the absence of any commercial or financial relationships that could be construed as a potential conflict of interest.
