# Supplementary material for: Research on Intelligent Identification of Pivoting Center and Smooth Processing of Test Data for Flying Flexible Joint
Source: Front Neurorobot. 2021 Apr 29;15:666285. doi: 10.3389/fnbot.2021.666285 (PMC8116692; doi:10.3389/fnbot.2021.666285)
Supplement: Supplementary file 1 [file Table_1.DOCX]

Table 1 comparison of pendulum center calculation results before and after data fairing (parabola)

| Before smooth processing | | after smooth processing | |
| --- | --- | --- | --- |
| xp | zp | xp1 | zp1 |
| 1.3451 | 6.4596 | 0.762 | 1.5636 |
|  |  | 0.458 | 0.9766 |
| -3.6879 | -4.934 | 0.0487 | 0.1329 |
|  |  | -0.3895 | -0.8348 |
| 3.4658 | 2.8112 | -0.8433 | -1.8392 |
|  |  | 0.7445 | 1.6646 |
| 1.7387 | -0.4697 | 0.3137 | 0.7034 |
|  |  | -0.0766 | -0.1668 |
| -2.8617 | -3.8671 | -0.3836 | -0.8596 |
|  |  | -0.598 | -1.3416 |
| 1.4189 | 0.5807 | -0.413 | -1.1362 |
|  |  | -0.3601 | -0.9591 |
| 5.5769 | 5.0588 | -0.1389 | -0.3626 |
|  |  | 0.2116 | 0.5736 |
| 0.7564 | 3.0488 | 0.7004 | 1.8843 |
|  |  | 0.4339 | 1.7495 |
| -2.5706 | -3.8186 | 0.4762 | 1.9012 |
|  |  | 0.2066 | 0.8266 |
| -5.1815 | -4.8698 | -0.3228 | -1.2683 |
|  |  | -0.7939 | -3.2088 |
| 81.8471 | -10.0718 | 0.2605 | 2.174 |
|  |  | -0.0232 | 0.7467 |
| -21.7729 | -1.2143 | -0.1834 | -0.3495 |
|  |  | -0.1765 | -1.1714 |
| -20.3057 | 3.0736 | 0.1224 | -1.3999 |
|  |  | -1.1196 | -1.6049 |
| -20.6393 | 3.0846 | -0.6301 | -0.9847 |
|  |  | -0.0603 | -0.1585 |
| -19.1293 | 5.128 | 0.5735 | 0.8328 |
|  |  | 1.2364 | 1.9153 |
| 259.2401 | 325.3455 | -1.2265 | -2.1285 |
|  |  | -0.5694 | -1.0032 |
| 130.4402 | 158.4533 | 0.0536 | 0.0806 |
|  |  | 0.6215 | 1.0801 |
| 71.1702 | 71.7063 | 1.1208 | 1.9709 |
|  |  | 0.762 | 1.5636 |
| 10.424 | -16.5635 | 0.458 | 0.9766 |

Table 2 comparison of swing center calculation results before and after data fairing (circular arc)

| Before smooth processing | | after smooth processing | |  |
| --- | --- | --- | --- | --- |
| xp | zp | xp1 | zp1 |  |
| -3.3133 | 0.0234 | 0.9017 | 2.6148 | |
|  |  | 1.6673 | 0.5784 | |
| 0.537 | 0.6628 | 0.5604 | 2.812 | |
|  |  | 1.6033 | 0.034 | |
| 3.6228 | 3.1509 | 0.2237 | 2.8892 | |
|  |  | 1.4476 | -0.4902 | |
| 1.5239 | 0.8435 | -0.1329 | 2.8091 | |
|  |  | 1.2519 | -0.9513 | |
| 1.5934 | 2.0389 | -0.5808 | 2.8605 | |
|  |  | 1.0961 | -1.6429 | |
| 2.2884 | 0.4126 | -0.8817 | 2.4848 | |
|  |  | 0.7657 | -2.0011 | |
| 0.5787 | -0.1712 | -1.0948 | 1.8849 | |
|  |  | 0.4271 | -2.105 | |
| -0.5611 | 0.7184 | -1.278 | 1.3815 | |
|  |  | 0.1361 | -2.2628 | |
| -1.5619 | -1.7836 | -1.374 | 0.8414 | |
|  |  | -0.1678 | -2.3243 | |
| -3.7164 | -5.898 | -3.1653 | -4.5981 | |
|  |  | -2.2548 | -6.9034 | |
| -9.9992 | -2.2006 | -1.9685 | -2.2596 | |
|  |  | -2.6311 | -0.5887 | |
| -1.1727 | -2.9234 | -1.325 | -2.7617 | |
|  |  | -2.5889 | 0.4779 | |
| -0.2831 | -0.2268 | -0.716 | -2.6279 | |
|  |  | -2.1874 | 1.1221 | |
| 1.2876 | 0.1207 | 0.3143 | -3.0385 | |
|  |  | -1.8179 | 2.516 | |
| 1.8575 | 2.581 | 1.015 | -2.2963 | |
|  |  | -0.8459 | 2.8209 | |
| 2.7471 | 1.3578 | 1.5257 | -1.2741 | |
|  |  | 0.1079 | 2.6114 | |
| -0.2831 | 0.3987 | 2.1021 | -0.7748 | |
|  |  | 0.7997 | 2.7502 | |
| 4.9155 | 1.7887 | 2.5617 | -0.2464 | |
|  |  | 1.4875 | 2.7485 | |
| -0.0607 | -0.894 | 2.9461 | 0.2822 | |
